# Ovotesticular differences of sex development: male or female? Case series

**DOI:** 10.1186/s13052-019-0660-8

**Published:** 2019-05-30

**Authors:** Maria-Grazia Scarpa, Arianna Lesma, Massimo Di Grazia, Waifro Rigamonti

**Affiliations:** 1 Unity of Pediatric Surgery and Urology, Institute for Maternal and Child Health - IRCCS Burlo Garofolo, via dell’Istria, 65/1, Trieste, Italy; 2 Unity of Pediatric Surgery, Department of Urology, San Raffaele Scientific Institute, Milan, Italy

**Keywords:** DSD, Ovotestis, Fertility

## Abstract

**Background:**

The choice of the sex of rearing in patients with ovotesticular differences of sex development (OT-DSD) is difficult. The final decision should be given by the patient himself or herself, but families’ opinion is not to neglect especially when the diagnosis is precocious and the patient can’t give the consent to medical or surgical procedures. How should we behave if the parents refuse to raise a child with genital ambiguity?

**Case presentation:**

We describe and comment on our multidisciplinary approach in three patients with neonatal diagnosis of OT-DSD. The families expressed a strong desire for that which concerned the sex of rearing of their babies in contrast to the International trend of “wait and see”. A specific counselling and a constant psychological support were given.

**Conclusions:**

Recent trends suggest of postponing surgery to involve the patient in the decision. Child’s well-being is the goal of therapy. When medical and psychological support is not able to force parents to accept a child suffering from genital ambiguity, we think that it is better to opt for reversible medical/surgical treatments rather than allowing patients to grow up within a family that does not accept them.

## Background

Ovotesticular difference of sex development (OT-DSD) is characterized by the simultaneous presence of testis and ovary in the same individual. It occurs between 3 and 10% of the total DSD [1]. In Europe, the most common karyotype is 46 XX (53%), followed by chromosomal mosaicism/chimerism (40%) and 46 XY karyotype (7%). *Matsui* reports that the most common gonadal combination is ovotestis and ovary (33.9%), followed by ovary and testis (24.2%), bilateral ovotestis (20.6%) and ovotestis and testis (16.4%). The gonadal combination of ovotestis and streak gonad occurs in only 1.2% of the cases [2]. Gonadal tumors occur between 2.6 and 4.6% of OT-DSD, more frequently in cases 46 XY [3]. Concerning Müllerian remnants, the possibility of degeneration is exceptional, although some Authors describe it [4]. A rigorous follow-up is therefore needed.

Gender assignment in newborns with OT-DSD represents a therapeutic challenge. According to the Chicago Consensus Statement on Management of Intersex Disorders, the factors that influence gender assignment include:

- Diagnosis
- Genital appearance
- Surgical options
- Need for life long replacement therapy
- Potential for fertility
- Views of the family
- Circumstances relating to cultural practise, [5].

Social and cultural aspects and family’s wishes about the sex of rearing are essential because the child well-being in the familiar and socio-cultural context must be the final goal of the treatment.

The recent current opinions are against a precocious operation. The patient consent is considered crucial. In this respect, what should be the behaviour of the surgeon? Our opinion is that the psychologist has a fundamental role in the DSD team decisions.

## Case presentation

We describe and comment our multidisciplinary approach in three cases of OT-DSD.

### Case series

**Fig. 1:**
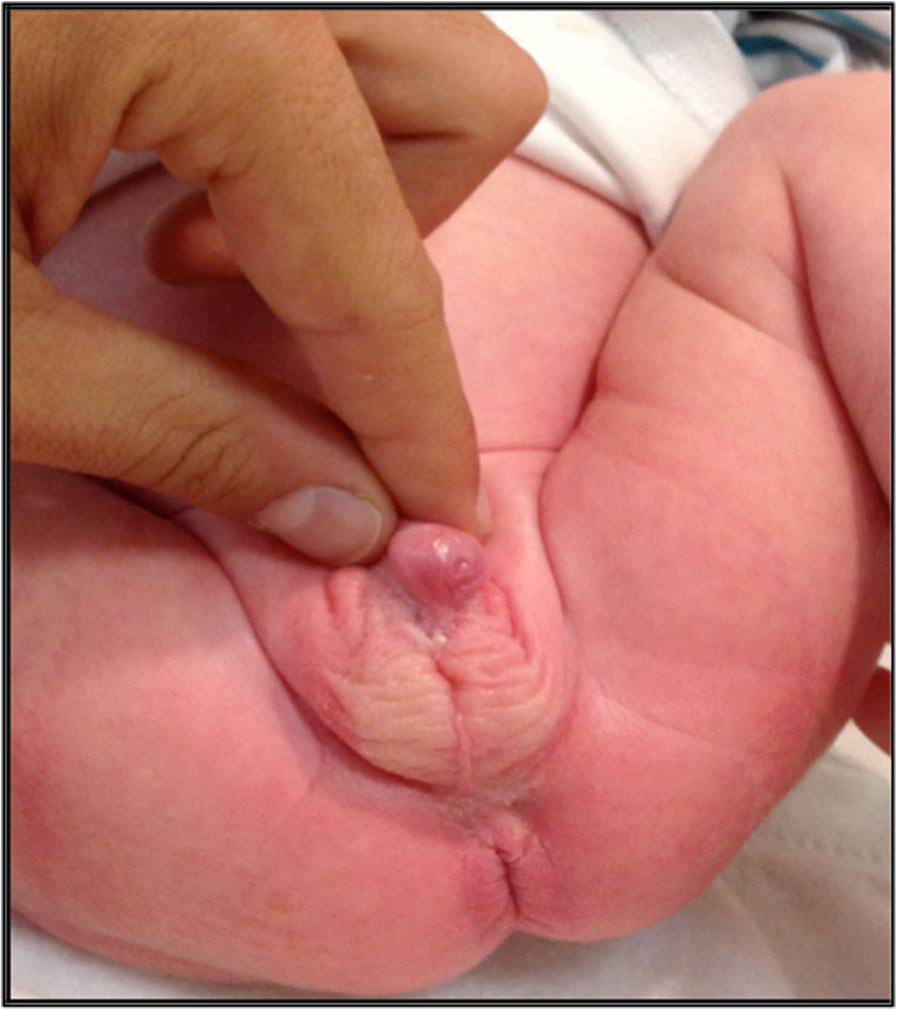
Phenotypic appearance of case 1

- *Case 1:* a newborn was transferred to our Institute for Maternal and Child Health “Burlo Garofolo” in Trieste for genital ambiguity. At birth the patient presented:

- Hypospadias without micropenis
- Penile curvature
- Impalpable gonads (see Fig. 1).

No familiar, gestational or perinatal problems were reported. Fetal sonogram assessed a male phenotype. Postnatal karyotype was 46 XY. Cytogenetic analysis on peripheral blood excluded a chromosomal mosaicism.

**Fig. 2:**
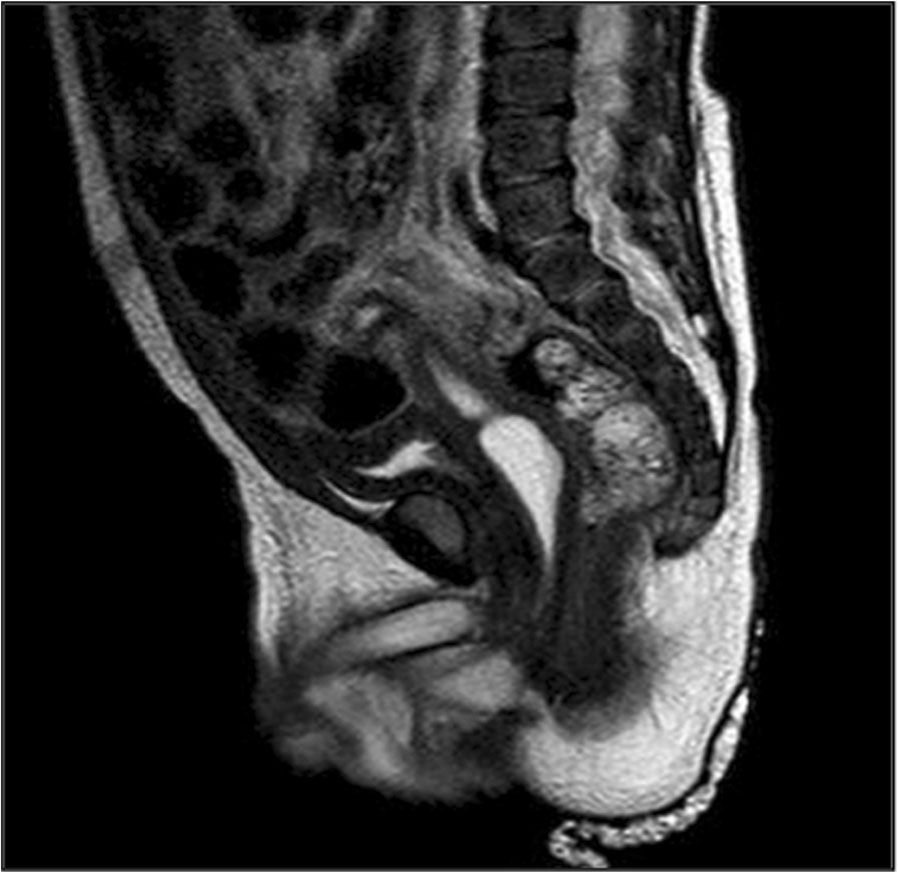
X-ray saggital image of MRI of case 1

**Fig. 3:**
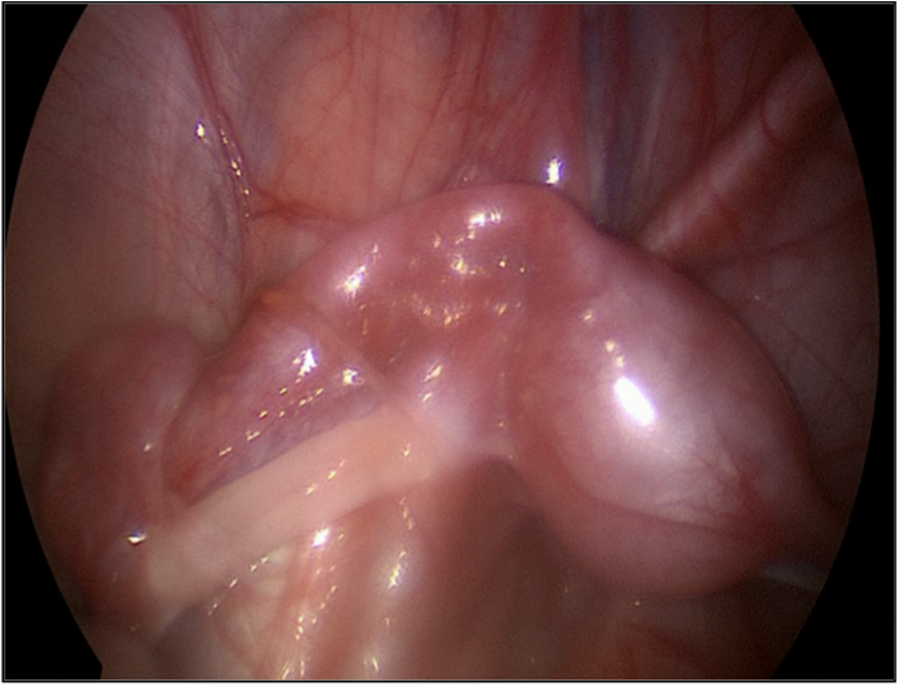
Intraoperative image of uterus, Fallopian tubev and streak gonad in case 1

**Fig. 4:**
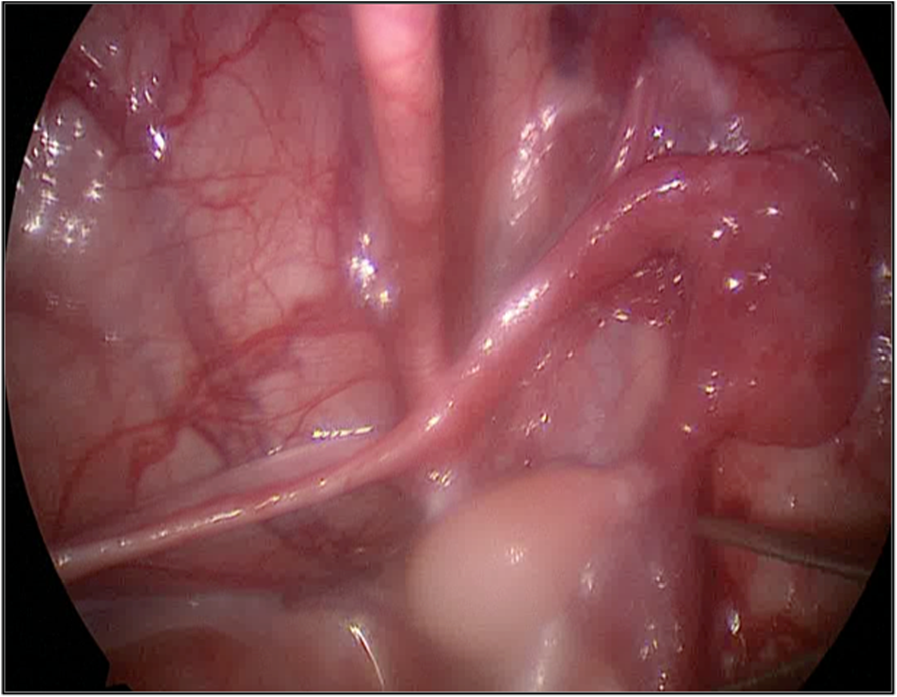
Intraoperative image of vas and testis in case 1

Stimulation with Human Chorionic Gonadotropin (hCG test) was positive: serum total testosterone level [ng/mL] pre-hCG was 0.68 ng/ml, post-hCG 1.23 ng/ml. Serum Anti-Müllerian Hormone (AMH), inibin B and dihydrotestosterone (DHT) level were normal. Uterus and vagina were identified on ultrasound scan and Magnetic Resonance Imaging (MRI) (see Fig. 2) and confirmed on laparoscopy. We found a streak gonad on the left side (see Fig. 3): its histological examination showed the presence of follicles; on the right side there were a vas deferens from the uterus and a testicle (see Fig. 4), this was confirmed through a biopsy. Socio-economic status of the family was low. Parents strongly wished a male baby. The counselling with the DSD team and the psychological consultations didn’t change family’s opinion about the sex of rearing. Orchiopexy and the first correction stage of the hypospadias were performed; Müllerian tissue was left in situ. The age at operation was 17 months. Last follow-up, at the age of 20 months, psychological examination confirmed that the child’s behaviour and its games were typically male.

- Case 2 was born at “S. Raffaele” Scientific Institute in Milan. Familiar, gestational and perinatal history was normal. The newborn presented:

- Enlarged clitoris
- Labial fusion
- Palpable gonads
- Single external meatus.

Postnatal karyotype was 46 XX, Fluorescent In Situ Hybridization (FISH) for Sex determining Region Y (SRY) was negative and cytogenetic investigations excluded a mosaicism. Ultrasound scan revealed the presence of the uterus with a cystic female-like gonad on the right side and a male-like gonad on the left inguinal position. Serum testosterone pre-hCG was 0.62 ng/mL, post-hCG 1.91 ng/mL. Serum AMH and Inibin B level were detectable. The family didn’t accept a baby with an indeterminate sex. A multidisciplinary discussion, which considered the views of the family, suggested a female sex of rearing. After surgery, at the age of twelve months, a right ovary and a left ovotestis were histologically confirmed: the testicular part was separated and removed from the ovary.

**Fig. 5:**
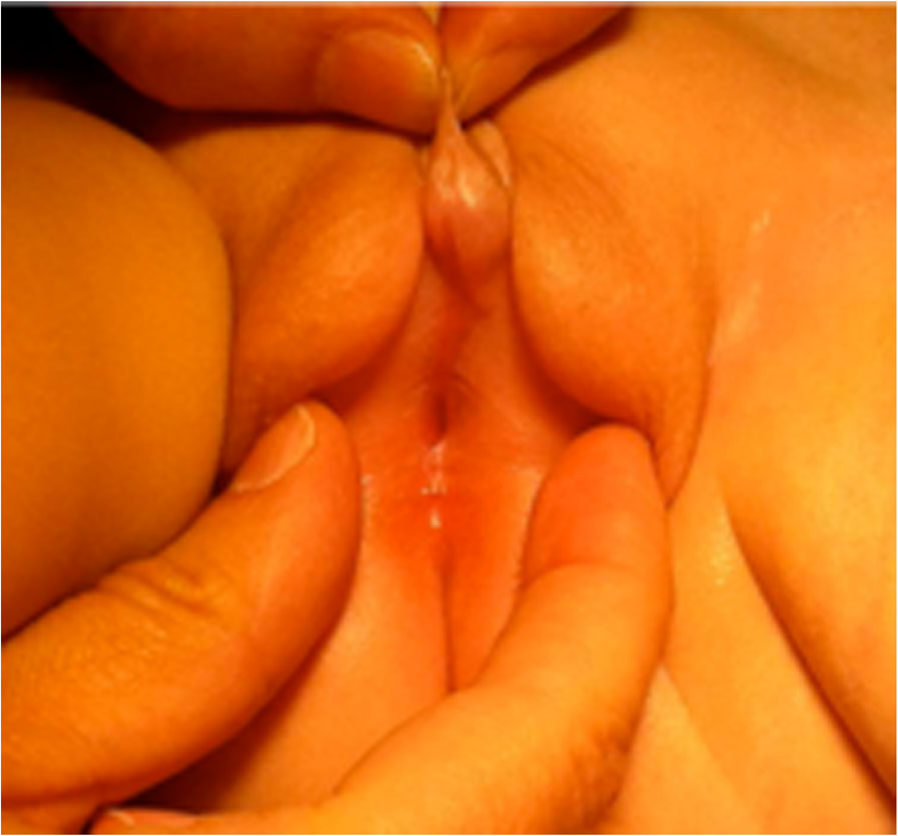
Phenotypic appearance of case 3, similar to the case 2

- Case 3 had the same external genital appearance of case 2 (see Figure 5) and differed from her because of the absence of a uterus and presence of bilateral ovotestis, histologically confirmed. Both testicular part were separated and removed from ovary. Array-based comparative genomic hybrizidation (Array-CGH) showed a duplication of paternal origin in the chromosome 17q24 which is a transcriptional enhancer of SOX9 gene. This mutation results in familial 46 XX DSD without any effect on the XY background [6]. Serum testosterone pre-hCG was < 0.1 ng/mL, post-hCG 0.76 ng/mL. Serum AMH and Inibin B level were detectable. The surgery was planned, respecting the family’s wishes on the female sex of rearing, after multisciplinary discussion and specific psychological counselling.

## Discussion

Most children with OT-DSD present ambiguous genitalia as newborns or infants. Rarely, OT-DSD has been detected later in individuals with female or male normal phenotype.

The ovarian tissue is usually normal and demonstrates follicular growth. The testicular tissue is often dysgenetic with hyalinization of the seminiferous tubules and poor germ cell development almost always resulting in infertility in patients assigned to the male gender [7].

Annual ultrasound surveillance is recommended for gonadal cancer risk. If precancerous lesions are suspected, more invasive procedures (MRI, cancer serum markers, biopsy) are mandatory.

Surgery is necessary after gender assignment and includes removal of gonads and internal ducts inappropriate to the sex of rearing and genitoplasty to construct the appropriate external appearance [2]. In our opinion the internal ducts might be left in situ: they can theoretically be useful for an eventual sex-reverse genitoplasty. The timing of surgery remains contentious [5]. According to recent trends, it is better to postpone surgery and maintain an indeterminate gender until the patient can participate to the decision.

Gender assignment is difficult to decide for OT-DSD patients. Families often ask for an early surgical solution to ensure child well-being within the family, the school and the society. What should the surgeon do in this case? Which kind of surgical consent form, should the families sign? We think that a multidisciplinary approach can define the best opportunity. All the treatments require excellent medical, surgical and psychological expertise. The fertility potential must be respected and a satisfactory result must be obtained. As far as our case series is concerned, in case 1 we removed the female gonad because of its macroscopic aspect of streak gonad with a potential tendency to degeneration. Although the fertility potential in a male OT-DSD is doubtful [8], the future presence of estradiol in developing ovarian follicles could inhibit spermatogonia development in contralateral seminiferous tubules. There is no evidence that prophylactic removal of asymptomatic Müllerian remnants is required [7]. If possible, we suggest leaving them in situ in male patients.

In cases 2 and 3 we maintained the ovarian part of the ovotestis for preserving a potential fertility even if in case 3 the uterus was not found. The families of the last two cases were strongly oriented to a female sex of rearing.

Sexual assignment in OT-DSD is a challenge: to date only few studies about gender dysphoria in this group of patients and no specific well coded guidelines exist. For appropriate management of these patients we refer to the general principles of the Consensus Conference of Chicago [5].

## Conclusion

We consider the important role of the psychologist within the multidisciplinary team, both for the role of family support, both to guide the choice on the sex of rearing.

Conservative treatment is the goal in the treatment of DSD, especially when the gender assignment is required during neonatal period.

Surgery must be as conservative as possible, granting the future possibility of a sex reverse genitoplasty and maintaining Müllerian structures in situ in a male patient.

Waiting for the pre-pubertal age is the best choice when an expert psychologist and a multidisciplinary team can support the family. In our opinion, when the parents refuse any explanation and support, child’s well-being is the most important right and the principal goal of the multidisciplinary DSD team. We think it is better a conservative surgery instead of leave the child growing in a non-acceptance’s atmosphere.

In such cases, we prefer a precocious surgery, considering dangerous “wait and see” in the following cases:

The family doesn’t accept a constant psychological support

Cultural or social issue are too deep

The family does not accept genital ambiguity: e.g. parents with psychological diseases, very low socioeconomic status.

Our future aims are:

- To collect new cases of OT-DSD and participate to multicenter studies
- To create a surgical consent form with a part reserved to the psychological interview.

## Data Availability

Data sharing not applicable to this article as no datasets were generated or analyzed during the current study.

## Abbreviations

AMH: Anti-Müllerian Hormone
DHT: Dihydrotestosterone
FISH: Fluorescent In Situ Hybridization
hCG: Human Chorionic Gonadotropin
MRI: Magnetic Resonance Imaging
OT-DSD: Ovotesticular Differences of Sex Development
SRY: Sex determining Region Y

## References

[1] Krstic ZD, Smoljanic Z, Vulkanic D (2000). True hermaphroditism: 10 years experience. Pediatr Surg Int.

[2] Matsui F, Shimada K, Matsumoto F (2011). Long-term outcome of ovotesticular disorder of sex development: a single center experience. Int J Urol.

[3] Pleskacova J, Hersmus R, Oosterhuis JW (2010). Tumor risk in disorders of sex development. Sex Dev.

[4] Farikullah J, Ehtisham S, Nappo S, Patel L, Hennayake S (2012). Persistent Müllarian duct syndrome: lesson learned from managing a series of eight patients over a 10-year period and review of literature regarding malignant risk from the Müllarian remnants. BJU.

[5] Hughes IA, Houk C, Ahmed SF, Lee PA (2006). LWPES1/ESPE2 consensus group: consensus statement on management of intersex disorders. Arch Dis Child.

[6] Vetro Annalisa, Dehghani Mohammad Reza, Kraoua Lilia, Giorda Roberto, Beri Silvana, Cardarelli Laura, Merico Maurizio, Manolakos Emmanouil, Parada-Bustamante Alexis, Castro Andrea, Radi Orietta, Camerino Giovanna, Brusco Alfredo, Sabaghian Marjan, Sofocleous Crystalena, Forzano Francesca, Palumbo Pietro, Palumbo Orazio, Calvano Savino, Zelante Leopoldo, Grammatico Paola, Giglio Sabrina, Basly Mohamed, Chaabouni Myriam, Carella Massimo, Russo Gianni, Bonaglia Maria Clara, Zuffardi Orsetta (2014). Testis development in the absence of SRY: chromosomal rearrangements at SOX9 and SOX3. European Journal of Human Genetics.

[7] Tran CN, Semins MJ, Epstein JI, Gearhart JP (2011). Ovotesticular disorder of sex development with mosaic 45, X/ 46, X, idic (Y) (q11.23) Kariotype and streak gonad. Urology (Pediatric Case Report).

[8] Shannon R, Nicolaides N (1973). True hermaphroditism with oogenesis and spermatogenesis. Aust N Z Obstet Gynaecol.

